# Abnormal Epigenetic Regulation of Immune System during Aging

**DOI:** 10.3389/fimmu.2018.00197

**Published:** 2018-02-12

**Authors:** Miriam G. Jasiulionis

**Affiliations:** ^1^Laboratory of Ontogeny and Epigenetics, Pharmacology Department, Universidade Federal de São Paulo, São Paulo, Brazil

**Keywords:** immune aging, epigenetics, DNA methylation, histones, environment, age-related diseases

## Abstract

Epigenetics refers to the study of mechanisms controlling the chromatin structure, which has fundamental role in the regulation of gene expression and genome stability. Epigenetic marks, such as DNA methylation and histone modifications, are established during embryonic development and epigenetic profiles are stably inherited during mitosis, ensuring cell differentiation and fate. Under the effect of intrinsic and extrinsic factors, such as metabolic profile, hormones, nutrition, drugs, smoke, and stress, epigenetic marks are actively modulated. In this sense, the lifestyle may affect significantly the epigenome, and as a result, the gene expression profile and cell function. Epigenetic alterations are a hallmark of aging and diseases, such as cancer. Among biological systems compromised with aging is the decline of immune response. Different regulators of immune response have their promoters and enhancers susceptible to the modulation by epigenetic marks, which is fundamental to the differentiation and function of immune cells. Consistent evidence has showed the regulation of innate immune cells, and T and B lymphocytes by epigenetic mechanisms. Therefore, age-dependent alterations in epigenetic marks may result in the decline of immune function and this might contribute to the increased incidence of diseases in old people. In order to maintain health, we need to better understand how to avoid epigenetic alterations related to immune aging. In this review, the contribution of epigenetic mechanisms to the loss of immune function during aging will be discussed, and the promise of new means of disease prevention and management will be pointed.

## Epigenetics: How the Genome Talks with the Environment

Epigenetics (epi = beyond) refers to the study of the heritable information on the chromatin beyond that given by the DNA sequence. Epigenetic marks, represented by different chemical groups added on both the DNA molecule and histone proteins, play key roles in the control of chromatin structure and function (1, 2). Among the most studied epigenetic mechanisms are DNA methylation and posttranslational histone modifications. The chromatin remodeling, the presence of structural and functional variants of histones, and the regulation by noncoding RNAs are additional epigenetic mechanisms working together with DNA methylation and histone modifications to maintain genome stability and control gene expression.

### DNA Methylation

In mammals, each cytosine in the context of a CpG dinucleotide is a potential site for the covalent addition of a methyl group, yielding 5-methylcytosine (5mC) (3). This reaction is catalyzed by DNA methyltransferases (DNMTs)—DNMT1, 3A, and 3B, which transfer the methyl group from *S*-adenosylmethionine (SAM) to the 5-carbon of the cytosine (5mC) (4). SAM, a component of methionine cycle, is considered the universal donor of methyl groups to various biomolecules, including DNA and histones (5). Different components coming from the diet, such as B6 and B12 vitamin, choline, and betaine act as cofactors in reactions taking part in methionine cycle, and the folate cycle is coupled to the methionine cycle, bringing up how cellular nutrient status may modulate epigenetic marks (6). In normal cells, most CpG-rich (CpG islands) gene promoters are unmethylated, making these genes permissive to transcription. Hypermethylated CpG-rich promoters are typically associated with gene silencing, since methylated CpGs can both impair the binding of transcriptional factors and recruit repressive complexes (7, 8). Regions, such as repetitive DNA sequences and transposons, present in high number in our genome, have CpGs heavily methylated in normal cells. The loss of methylation in these regions favors homologous recombination and the expression of undesirable elements, contributing to chromosomal instability (9). The technological advances in genome-wide chromatin profiling have revealed that in fact the role of DNA methylation in gene regulation depends on its position and context. While in promoters it is associated with transcriptional silencing, in other regions it can modulate enhancer activity and splicing (10). The methylation at gene bodies is frequent in ubiquitously expressed genes and correlated with transcriptional activation (11). The tissue-specific DNA methylation seems to occur not at CpG islands, but at regions of lower CpG density about 2 kb distant from CpG islands, named CpG island shores (12). In addition, other non-CpG sites were more recently described as sites for DNA methylation in humans, such as CHG and CHH sites (where H is A, C, or T) (13), but the mechanisms involved in this process are still unknown. The complexity of the covalent modification of DNA has additionally increased with the recent identification of a new family of enzymes known as ten-eleven translocation 1-3 (TET1-3), which are able to oxidize 5mC to 5-hydroxymethylcytosine (5hmC) in a reaction that generates other intermediates (5-methylformylcytosine and 5-carboxylcytosine) (14, 15). These modified bases may be excised by thymine DNA glycosylases (TDG) and, through the base excision repair (BER) process, yield demethylated cytosines (16), which makes this process a mechanism of active DNA demethylation. The DNA can be also actively demethylated by the deamination of 5mC and 5hmC by the activation-induced deaminase/apolipoprotein B editing complex enzymes, followed by BER/TDG activity (17). Alternatively, 5hmC can be passively demethylated during DNA replication, since it is not recognized by DNMT1, yielding in this position non-methylated cytosine in the newly synthesized DNA strand.

### Histone Modifications

In the nucleosomes, not only the DNA molecule but also histone proteins carry chemical modifications, which are fundamental for chromatin-dependent gene regulation (18). Several posttranslational histone modifications (PTMs) regulate the chromatin structure, by affecting inter-nucleosomal interactions, and recruit proteins and complexes that influence not only the gene transcription but also mediate processes, such as DNA replication, DNA repair, alternative splicing, and recombination (19). A large number of proteins acting as writers, erasers, and readers have been described as components of histone modifier machinery, targeting all core histones H2A, H2B, H3, and H4, and the linker H1 histone, which have their amino acids subjected to covalent modifications mainly in the *N*-terminal tails. Among these modifications are acetylation, phosphorylation, methylation, ubiquitylation, sumoylation, and ADP ribosylation (19). By decreasing the positive charge of histones, acetylation and phosphorylation weaken interactions between histones and DNA, facilitating transcription machinery to access the DNA. Histone methylation occurs mainly on lysines and arginines, which can be, respectively, mono-, di- or trimethylated, and mono- and di-methylated, resulting in a high level of complexity regarding their effects. For example, high levels of trimethylated H3K4, H3K36, and H3K79 are associated with actively transcribed chromatin, while methylated H3K9, H3K27, and H4K20 are associated with transcriptionally inactive chromatin (20). The covalent attachment of the large ubiquitin molecule changes the nucleosome conformation, affecting both intra-nucleosomal interactions and interactions with effector proteins. Sumoylation involves the addition of small ubitiquin-like molecules to histones, and has been associated with repressive functions. Histone mono- and poly-ADP ribosylation has been correlated with a relaxed chromatin state. The number of possible histone modifications has increased with the continuous identification of novel histone PTMs, such as lysine propionylation (21, 22), butyrylation (21), crotonylation (23), succinylation, and malonylation (24), coupling cell metabolism with chromatin structure and function. It is important to keep in mind that a single histone mark is not responsible for the final effect on chromatin, but rather the combination of all marks in a chromatin region defines the biological outcome (25, 26). Besides that, there is interplay of DNA methylation, histone modifications, and nucleosome positioning, and the outcome is a result of the sum of these interactions.

### The Plasticity of the Epigenetic Marks

Although presenting the same genome, each cell type in the same individual has a specific group of epigenetic marks, named epigenome. Epigenetic marks are established during embryonic development and transmitted through mitosis, stabilizing gene expression programs, and defining cell-type identities and function (27). In addition to their role in cell differentiation, these marks are fundamental to X chromosome inactivation in females and genomic imprinting during development (28, 29). Although being relatively stable over time, epigenetic marks can change dynamically in response to cellular conditions and environmental cues (2, 30, 31). Recent studies have shown that the methylation patterns determined by the binding of factors on DNA motifs are less responsive to environment within the lifetime of an individual, and that these patterns would persist across generations (32, 33). However, other DNA regions would have their epigenetic marks more susceptible to internal and external environment, such as stress, smoke, drugs, hormones, circadian rhythms, and metabolic variations caused by diet. In this way, the environment can modulate the epigenotype, and consequently the phenotype, being decisive to direct to health or disease states. In fact, many studies have shown the relation between epigenetic alterations and a wide variety of diseases, including aging-related diseases (34–36).

## Changes in Epigenetic Marks During Aging

During the aging of an organism, there is a gradual decline of normal physiological functions. In humans, these include decreased immune function, chronic inflammation, sarcopenia, and most importantly increased susceptibility to diseases, such as cancer, cardiovascular disorders, and metabolic and neurodegenerative diseases. Although systemic, these phenotypes are a result of alterations in different cellular processes, such as DNA damage response, mitochondrial and proteasome function, and cell death regulation (37–40). At the molecular level, transcriptional dysregulation is observed with aging, resulting in gene expression changes (41, 42). Epigenetic alterations are important contributors to these changes in the aged transcriptome, and are known as “epigenetic drift” (43–45).

### DNA Methylation

Regarding DNA methylation, a progressive global hypomethylation occurs invariably with advanced age (46). Repetitive DNA sequences normally silenced by epigenetic marks become expressed, being at least partly responsible for the well-characterized loss of heterochromatin observed during aging (47, 48). An age-dependent hypomethylation of specific gene promoters, such as *IL17RC*, occurs and induces their transcription (49). At the same time, some gene promoters become hypermethylated and abnormally silenced (50–52). Regarding 5hmC, it was shown that although the global level of 5hmC in the brain increases with aging both in mice (53) and humans (54), it decreases in other tissues, such as blood (55).

Besides the epigenetic drift, which is stochastic non-site-specific changes in DNA methylation that contributes to variability during aging, DNA methylation signatures in specific CpGs, both tissue-specific and present in several tissue types, were identified as associated with chronological age (56). Although age-related DNA methylation alterations are more frequent in CpG islands, tissue-specific changes occur in other genomic regions (57). In a comprehensive study of DNA methylation, Yuan and colleagues (58) showed that besides hypermethylated CpG islands, a great number of age-related differentially methylated regions fell into open sea (regions of megabase extension characterized by low CG content) or shore/shelf regions, which were found hypomethylated with age. These authors have also identified large age-associated hypomethylated blocks, similar to those described associated with cancer (59). Based on the genome-wide methylation profile of whole blood from 656 individuals spanning a wide age range, a quantitative model was built to determine the rate at which an individual’s methylome ages, and was shown to represent a strong and reproducible mean to discriminate relevant factors in aging (60).

### Histone Modifications

The global DNA hypomethylation observed during aging was shown to be associated with changes in histone modification patterns (61, 62). Changes in the activity, function, and abundance of enzymes of the epigenetic machinery are present with aging (63, 64). Genes identified as hypermethylated in blood cells during aging were associated with the presence of bivalent chromatin domains in embryonic stem cells and with the repressive histone marks H3K27me3 and H3K9me3 in differentiated cells (65–67). A global loss of histones, as well as an imbalance of activating and repressive histone marks, occurs with age (68, 69). For example, a diminished content of acetylated H3K9 (70) and trimethylated H3K27 (71) was described in aged cells. Reduced levels of H3K9me3, which can be a result of the downregulation of SUV39H1/2 (72), were found with age in human and murine tissues and cells and seem to contribute to the loss of heterochromatin (72, 73). An age-decrease in the expression of HP1 (74) and DNMTs (75) could favor DNA demethylation in the heterochromatin. Another alteration that could contribute to a more opened chromatin state is the increased level of H4K16Ac with replicative age, as described in human fibroblasts in culture (76). H4K16 is among the targets of the NAD^+^-dependent histone deacetylase SIRT1, which is associated with aging extent and genome maintenance in different organisms (77).

While the levels of the canonical histones decrease during aging, alterations in the replication-independent incorporation of histone variants occur during aging. The replication-independent histone variant H3.3 becomes more abundant with age in general, not just in non-replicating cells, such as neurons (78). Again, this could favor a chromatin state more accessible to the transcription machinery. Another replication-independent histone variant that seems to be linked to aging is the H2A.Z, since H2A.Z knockdown fibroblasts were shown to develop premature senescence (79). The H2A variant macroH2A is characteristic of senescence-associated heterochromatin foci, heterochromatin regions over proliferation-promoting genes in senescent cells (80). An age-dependent increase in the macroH2A level was described both during replicative senescence in cultured human fibroblasts and in many tissues of aged mammals (81).

### Effects of the Environmental and Lifestyle Factors on Epigenetic Changes and Aging

A classical study by Esteller’s group (82) showed significant differences in epigenetic marks in old monozygotic twin pairs compared to very young twin pairs, which presented these marks indistinguishable. More interestingly, those old twin pairs that had spent less of their lifetime together and/or had a more different natural health-medical history were those presenting the greatest differences in epigenetic marks. Studies with human population have shown that genetic factors cause no more than 20–30% of the differences observed in the lifespan of identical twins, the epigenetic drift being the main responsible for variation during the lifetime (83, 84). These and other studies (85–88) illustrate how epigenetic marks change with aging and are under the effect of environment. As a whole, these alterations change the chromatin accessibility, resulting in abnormal gene transcription and genomic instability, and have been proposed to be key regulators of the aging process, contributors to the development of age-related diseases and even predictors of the chronological age (52, 89–93). Age-related changes in multiple CpG sites across the genome were shown to accurately predict the biological age of an individual. This epigenetic clock has been shown a potential biomarker of aging in humans and associated with several aging-related disease phenotypes (60, 90, 94, 95). Epigenetic age assessed in blood was able to predict, independently of chronological age, all-cause mortality in different cohorts, including different racial/ethnic groups (93, 95–97).

It is important to emphasize that the epigenome acts as a molecular interface between the genome and the environment. In this way, the lifestyle, including diet habits, exercises, life stressors, smoke, substance abuse, chemical exposition, among others, could alter the epigenetic landscape, affecting the chromatin structure and function, and, consequently, favoring the development of aging-related disease phenotypes. Exercise and nutritional habits remodel epigenetic marks in human skeletal muscle and adipose tissue (98–100). The effect of exercise on the improved cardiorespiratory fitness and running performance, as well as on the decreased low-density lipoprotein levels, was accompanied by a widespread demethylation of CpG islands, opposed of the methylation changes observed during aging (101, 102). Several studies have demonstrated the adverse effect of smoke associated with changes in epigenetic marks. Prenatal smoke exposure affects DNA methylation of blood cells from children of smoking mothers (103). Epigenetic alterations caused by chronic cigarette smoke sensitize bronchial epithelial cells to malignant transformation (104). Tobacco smoking may induce DNA methylation alterations in cell types of both the innate and adaptive immune system (105). Offspring DNA methylation alterations were associated with maternal alcohol consumption (106). The turnover of histones and histone variants was shown to be affected by the alcohol exposure in rats (107). Many of these effects of the environment on aging involve oxidative stress, both in humans and animal models. Although severe acute or chronic stress exposure accelerates aging by favoring error accumulation due to exhausting defense mechanisms, moderate stress has shown to delay aging process by activating defense mechanisms to prevent and/or eliminate errors (108). In the last years, several studies have demonstrated the relation between cellular stress and epigenetic alterations (104, 109–114). Reactive oxygen species (ROS) lead to oxidized DNA lesions that can contribute to DNA methylation alterations. One of the major DNA oxidative damage products is 8-hydroxy-2′-deoxy-guanosine that impairs binding of DNMTs and methyl-CpG binding proteins to DNA (115). In addition, ROS may interfere with TET-mediated DNA demethylation (116). SAM availability can also be decreased by the depletion of glutathione (GSH) because of redox status, inhibiting all methylation reactions (117). Sirtuins play important role in response to a variety of stresses, such as oxidative or genotoxic stress and are crucial for cell metabolism. ROS can both induce DNA damage and SIRT1 relocation to these damage sites, for where SIRT1 recruits other epigenetic machinery components, such as DNMTs and polycomb proteins in order to silence these regions. O’Hagan and coworkers (110, 112) showed that this process could result in stable aberrant epigenetic and gene transcription changes, similarly to alterations observed in cancer. In murine embryonic mesenchymal fibroblasts, increased levels of hydrogen peroxide induce SIRT1 to relocate from repressed DNA sequences to DNA breaks to promote repair, resulting in transcriptional changes that parallel those in the aging mouse brain (118). By responding to environmental stress, sirtuins promote cell survival and, as a result, increase replicative and chronological lifespan. Although not clearly established in mammals, the association of sirtuins with aging and lifespan is suggested by the overexpression of SIRT1 in murine tissues during caloric restriction (CR) (119), the requirement of Sirt1 to the increased physical activity and extended lifespan during caloric restriction (120), and the improved health and survival of mice submitted to a high-calorie diet after resveratrol treatment, which activates Sirt1 (121). Several studies in different model organisms show the role of sirtuins in lifespan extension by CR (122–125), and evidences indicate that epigenetic mechanisms have crucial roles in this process (126, 127). In this context, new and known compounds have been tested as “CR mimetics,” including sirtuin-activating compounds, such as resveratrol (128). Compounds inhibiting histone acetylation, such as spermidine, also extend lifespan (129).

As mentioned before, ROS may modify the TET-mediated DNA demethylation (116). Both the increase in endogenous antioxidants and caloric restriction were shown to impair the increase in 5hmC levels in murine aged brains (130). The demethylase activity of TET enzymes can be stimulated by nutrients, such as ascorbic acid (131, 132). Since the activity of many epigenetic enzymes depend on intracellular levels of essential metabolites (methionine, iron, ketoglutarate, NAD^+^, acetyl-CoA, SAM), the cellular metabolism controls epigenetic modifications and may regulate longevity (133, 134).

In another aspect, studies in human cohorts have shown that life stressors, in special during early development, can induce lasting epigenome alterations (135–139). Stress and glucocorticoids may induce long-lasting changes in DNA methylation both at the genome-wide level and within selective gene loci, as observed both in humans and rodent models (140–142).

## Epigenetic Regulation of the Immune System

An important characteristic of the immune system is its adaptive capacity to recognize self from non-self to protect the organism in response to environmental signals of different types and duration, such as potentially pathogenic agents and substances. Several immune cell populations act against potentially hazard environment by both innate and adaptive mechanisms, and their functions depend on highly controlled regulation of hematopoietic cell differentiation. Increasing number of studies have demonstrated the crucial role of epigenetic mechanisms in the development and differentiation of immune system, as well as in related pathologies (143–145). With age, the immunocompetence becomes compromised and this has been linked to the repression of immune cell differentiation genes along with the activation of autoimmunity genes because of DNA methylation alterations (49, 146–148).

### Innate Immune Cells

The innate immune system, consisting of macrophages, neutrophils, dendritic cells (DCs), and natural killer (NK) cells, is the first response to pathogenic agents. Macrophages and DCs are professional antigen-presenting cells (APCs) able to capture antigens for processing and presentation to lymphocytes. When activated, resident macrophages can act directly by destroying their targets or indirectly *via* initiating an acute inflammatory response by producing cytokines, chemoattractants, and inflammatory mediators, and recruiting neutrophils, monocytes, and DCs (149). Activated macrophages release different factors in response to the extracellular environment, being able to acquire functionally distinct phenotypes, classic M1 and alternative M2. Activated M1 macrophages are induced by the cytokine interferon-gamma (IFN-γ) and bacterial products and have a pro-inflammatory profile, playing an important role in host defense. Differently, M2 macrophages are induced by interleukin-4 and -10 (IL-4 and IL-10) and helminthic products and have an anti-inflammatory profile, promoting tissue repair. Since mature cells of the immune system have to rapidly respond to pathogens, the contribution of epigenetic mechanisms to the regulation of genes involved in these responses has been substantially described. In this context, epigenetic mechanisms were shown to be involved in the modulation of macrophage polarization, mainly by histone marks present in enhancers of specific genes (150). The first study showing the epigenetic regulation of inflammation was that by Saccani and Natoli (151). They demonstrated the induction of inflammatory cytokines, such as IL-8 and macrophage inflammatory protein 1-alpha (MIP-1α), by the loss of H3K9 methylation at the promoter regions after exposing cultured human monocyte-derived DCs to bacterial endotoxin lipopolysaccharide (LPS). Innate immune cells have a degree of specificity by presenting pattern recognition receptors (PRRs) to recognize damage- or pathogen-associated molecular patterns in non-infectious substances or microbes, respectively (152). Recent evidences indicate that, different from previously believed, cells of innate immune system may keep a memory of past stimulations, named “trained immunity,” changing the response upon new stimuli and becoming able to respond to a larger number of microbes than the initial agent (153, 154). This immunological memory involves changes in transcriptional programs by reprogramming epigenetic marks. For example, metabolic changes in monocytes activated by β-glucan from *Candida* are associated with increased levels of the active histone marks, H3K4 trimethylation, and H3K27 acetylation, leading to increased production of IL-6 and TNF cytokines, inflammation, and “trained immunity” (155). Macrophages restimulated with LPS induce an attenuated inflammatory response, although maintaining an intact antimicrobial response. Foster and colleagues (156) showed that genes involved in LPS-tolerance lose the active histone marks H3K4me3 and H4Ac in their promoters during restimulation with LPS, while non-tolerizeable genes maintain these active marks after a secondary challenge with LPS, correlated with a permissive gene transcription. Epigenetic mechanisms also regulate the differentiation of human monocytes into DCs under specific stimuli. For example, the observed increased expression of CD209 during differentiation was shown to be a result of the acquisition of H3K9Ac and loss of H3K9me3, H4K20me3, and DNA methylation in its promoter (157).

### T Lymphocytes

The age-dependent deterioration of the immune system, named immunesenescence, is accompanied by alterations in epigenetic marks. Kuwahara and colleagues (158) showed that CD4 T-cell senescence and cytokine homeostasis is controlled by the maintenance of histone acetylation on the *Bach2* locus promoted by the binding of menin. In addition, the increased genomic instability in the thymus with age is associated with a loss of heterochromatin marks, including H3K9me3 with corresponding reduction in SUV39H1 expression (159). The senescence seems to be also activated by DNA hypomethylation since the hypomethylation is observed in senescing but not in immortalized cells (160), and the DNA methylation inhibition leads immortal cells to cell arrest (161).

Cells from the innate immune system present antigens to both B and T lymphocytes, activating them to proliferate and differentiate into effector cells. APCs activate T cell receptor and costimulatory molecules of naïve T cells, initiating T cell differentiation by the activation of the nuclear factor of activated T cells and production of interleukin-2 (IL-2). IL-2 orchestrates the molecular switch of transcriptional programs of immune-responsive genes in response to T cell activation. Naïve and resting CD4^+^ T cells do not express IL-2, but this cytokine is expressed in T cells under antigen stimulation. Murayama and colleagues (162) showed that demethylation of a single specific CpG site in an enhancer region is a prerequisite for IL-2 transcription and, more interestingly, that this epigenetic change constitutes a memory that CD4^+^ T cells encountered the antigen.

Peptide antigens are presented by APCs to T cells in the context of the major histocompatibility complex (MHC) molecules. Cytotoxic T cells, expressing CD8, recognize antigens presented by normal cells in the context of MHC class I molecules, being able to directly destroy the infected cells. Activated CD8^+^ T cells have increased levels of H3Ac at the *IFN-γ* promoter and enhancer, modification that is maintained through memory CD8^+^ T cells, and permits a quicker and stronger cytotoxic response to additional antigen stimulation (163). MHC class II are the MHC molecules involved in the antigen presentation to CD4^+^ helper T cells. The class II transactivator (CIITA) is a key factor controlling the expression of MHC-II, and both CIITA expression and CIITA-dependent MHC-II expression are epigenetically regulated (164). Analysis of chromatin accessibility in PBMCs identified memory CD8^+^ T cells as the subpopulation with the most profound chromatin remodeling with aging (165, 166).

After antigen recognition, depending on cytokine environment, naïve T lymphocytes differentiate into effector T “helper” (Th1, Th2, and Th17) or regulatory (Treg) CD4^+^ T cells, and coordinate specific immune responses by producing distinct sets of cytokines (167). The differentiation toward a Th1 profile is induced by IFN-γ, IL-12, or IL-15, whereas differentiation toward the Th2 profile—by IL-4, IL-10, or IL-13; both pathways involve the regulated expression of multiple effector genes. Transforming growth factor beta and IL-6 are responsible for inducing naïve T cell differentiation into Th17 cells. The CD4^+^ T cells differentiation into these different profiles is tightly regulated to assure specific cytokine signatures and changes in the epigenetic marks are fundamental to complete this process. The *IFNG* promoter, hypermethylated in human naïve T cells, becomes demethylated during the differentiation into Th1 profile (168). Specific histone marks were identified across the *IFNG* locus, where H4Ac and H3K4me3 are present in Th1 cells and H3K27me2 and H3K27me3 in Th2 cells (169). Naïve and Th1 cells present *IL-4* promoter highly methylated, while Th2 cells have the intron 2 of *IL-4* partly demethylated (170). Th17 cells are characterized by the expression of IL-17 cytokine and RAR-related orphan receptor C (RORC) transcription factor. The demethylation of both *IL-17A* and *RORC* loci correlates with gene expression in human Th17 cells (171), and the active histone marks H3Ac and H3K4me3 were found in the *IL-17* locus (172). The demethylation of *Foxp3* locus, as well the hyperacetylation of histones, was shown to be important to maintain the stable expression of forkhead box P3 (FOXP3) and stabilize the regulatory phenotype in Treg cells (173, 174).

### B Lymphocytes

After binding to an antigen and be induced by T helper cells, B cells differentiate into antibody-secreting plasma cells. Antibodies bind to the specific antigen, leading to a better recognition and destruction of the pathogen (such as bacteria, virus, and tumor cells) by activating complement and/or interacting with lytic cells. During B cell differentiation, lineage-specific genes are expressed, whereas genes related to multipotent progenitors and alternative lineages are repressed. Complex epigenetic regulatory mechanisms coordinate B cell differentiation and function, including monoallelic V(D)J rearrangement and antibody diversity (175–178). A key transcriptional factor involved in B cell commitment is paired box 5 (Pax5) that, besides having its expression regulated by epigenetic mechanisms (179, 180), recruits chromatin-modifying proteins to regulate the expression of its targets. For example, *CD79a* gene promoter, hypermethylated in the progenitor stage, becomes demethylated during early stages of B cell differentiation, followed by the action of histone acetyltransferases recruited by Pax5, allowing gene expression (181). Pax5 can also interact with chromatin-modifying enzymes to repress genes specific for other lineages (182). V(D)J rearrangement and antibody diversity are necessary for the production of effective antibodies and require the activation-induced cytidine deaminase (AID), expressed by B cells at specific stages of differentiation. In naïve B cells, *AID* gene promoter is hypermethylated and the gene is not expressed. Upon B cells activation, *AID* gene becomes demethylated and acquires increased levels of the active histone mark H3Ac (183). The acquisition of this histone mark in active promoters and distal enhancers is also crucial for gene expression changes occurring during the differentiation of B cells to plasma cells (184). Blimp-1, a transcriptional repressor that maintains plasma cell identity, has its expression epigenetically induced and epigenetically suppresses the expression of mature B cell genes by recruiting histone modifiers (185, 186). After V(D)J rearrangement and antibody diversity processes, B cells can differentiate into memory B cells, which acquire additional epigenetic marks beyond those acquired during B cell activation (187). Different epigenetic modifications, as well as epigenetic enzymes, such as enhancer of zeste homolog 2 (188), histone acetyltransferase monocytic leukemia zinc finger protein (189), and DNMT3a (190), are observed in resting and activated B cells, and indicate that the memory B cell epigenome could favor a faster and more efficient activation than that of naïve cells.

## Contribution of Epigenetic Alterations to Immune Aging

Age-associated defects are observed in all cells from the immune system, affecting their activation and cytokines production.

### Innate Immune Cells

Regarding the innate immune system, many immune responses decrease during aging, but at the same time hyperreactivity of some responses are also observed (191). Epigenetic alterations seem to affect the monocyte differentiation with age, since older hematopoietic progenitor cells (HPCs) present hypomethylation of differentiation-related genes compared to progenitor cells from umbilical cord blood (192). It could be related to the reduced pluripotency and decreased potential of differentiation of HPCs from older donors (193). At the same time, older HPCs presented *de novo* methylation of a subset of genes associated with the Polycomb repressive complex that could contribute to the reduced phenotypic plasticity of aged stem cells (192). Indeed, epigenetic dysfunction could be a precursor to hematologic disease in elderly individuals (194). In macrophages, epigenetic mechanisms contribute to the decreased expression of MHC-II observed with age (195). Although the number of NK cells increases in older individuals, their cytotoxic activity decrease, and DNA methylation regulation of IFN-γ and IL-2 seems to contribute to this defected function of NK cells (196). Aging is well characterized by an imbalance between inflammatory and anti-inflammatory responses, where increased levels of inflammatory mediators, such as IL-6 and tumor necrosis factor-alpha (TNF-α), are observed even in the absence of acute infection or other physiologic stress (process known as “inflammaging”) (197). TNF-α has its expression increased during aging linked to its promoter demethylation (198). This epigenetic alteration contributes to the increased levels of TNF-α and also IL-1α (199, 200), which initiate the low-grade inflammation associated with resting neutrophils from aged donors. A major cause of worldwide morbidity in the elderly is the age-associated inflammatory lung disease (201). In this context, promoter hypomethylation of inflammatory genes, such as toll-like receptor 2, carnitine *O*-acetyltransferase, and coagulation factor III, were associated with decreased lung function (202). Zinc is a micronutrient crucial for the development and function of immune system, and its deficiency, frequently observed during aging, contributes to a wide range of immune defects (203), including an enhanced inflammatory response by inducing *IL-6* promoter demethylation (204). Using the C-reactive protein (CRP) as an inflammatory biomarker, Ligthart and coworkers (205) performed a meta-analysis of epigenome wide association studies of DNA methylation on chronic low-grade inflammation. In this study, the authors demonstrated that several inflammation-related CpG sites were associated with the expression of nearby genes, and that many of these CpGs presented association with cardiometabolic phenotypes and incident coronary heart disease. Among these genes is *AIM2*, important in innate immune response since it takes part of host defense mechanisms against bacterial and viral pathogens, and that was found hypermethylated and expressed at low levels in samples with low levels of CRP.

### T Lymphocytes

The involution of thymic structure and function, characterized by a reduced number and functional defects of thymic naïve T cells, is other process contributing to the immune aging (206). By analyzing the methylome of CD4^+^ T cells from newborn and centenarian individuals, Heyn and coworkers (207) showed these immune cells present the same DNA methylation changes that are observed in other tissues during aging, a global DNA hypomethylation and a higher variability of DNA methylation. Later, by an integrated analysis of transcriptome, methylome, and miRNAome in the same CD4^+^ T cells, Zhao and colleagues (148) found a potential relationship between gene transcription and DNA methylation for age- or immune-related genes, indicating the involvement of DNA methylation in the transcription regulation related to the development and functions of T cells in aging. Mice with a heterozygous *Dnmt1* null mutation have hypomethylated DNA and showed to be phenotypically normal, but presented immune senescence and developed early autoimmunity compared with normal mice of the same age (208). By analyzing naïve CD4^+^ T cells from 74 healthy 19- to 66-year-old individuals, Dozmorov and colleagues (209) identified sites hypomethylated with age presenting T cell-specific enrichment in active enhancers marked with H3K27Ac and H3K4me1, suggesting a progressive age-associated shift in T-cell epigenomes toward pro-inflammatory and T cell activating phenotype that could contribute to increased autoimmunity with age. It was also shown that aged individuals, who have higher levels of autoantibodies, have T cells presenting demethylation and overexpression in the same genes demethylated and overexpressed in T cells from lupus patients (146). The progressive loss of the costimulatory molecule CD28 in CD4^+^ T lymphocytes during aging is associated with impaired immune response. Recently, a unique DNA methylation landscape was described in CD28^null^ T cells, leading to the expression of inflammasome-related genes (210). Other recent study found two CpG sites present in the promoter region of *KLF14*, involved in CD4^+^ T cell differentiation *via* suppression of FOXP3, that exhibit stable methylation early in life and a rapid increase late in life in peripheral whole blood, monocytes, and isolated CD4^+^ T cells (211). Dysfunctional Treg cells have been considered to be contributors to immune senescence and increased susceptibility to age-associated diseases by suppressing T cell responses. Garg and colleagues (212) showed that the high number of Treg cells observed in aged mice is associated with hypomethylation of the upstream *FoxP3* enhancer, resulting in its increased expression. They also demonstrated that Treg cells from aged mice release more IL-10, are more efficient in downregulating the costimulatory molecule CD86 on DCs, and modulate the extracellular redox environment, suppressing T cells proliferation.

Immune senescence is also characterized by a loss of naïve and central memory cells and an expansion of effector memory cells within the CD8^+^ T cell compartment. A shift toward more differentiated state of chromatin openness was observed in naïve and central memory cells from older individuals, as well a loss of chromatin accessibility at gene promoters mediated in part by the loss of nuclear respiratory factor 1 (NRF1) binding in aged naïve cells (213). By analyzing PBMCs methylation data set in an Italian population, Horvath and colleagues (214) showed that the centenarians are younger than expected based on their chronological age. McEwen and colleagues (215), by examining one of the highest old-age life expectancies populations from Costa Rica (Nicoyans), found this population to possess a significant higher abundance of predicted CD8^+^ T naïve cells and a lower abundance of estimated CD8^+^ T memory cells compared with non-Nicoyans, suggesting a younger immune cell profile. In addition, they showed a lower variability in the DNA methylation in Nicoyans compared with non-Nicoyans as an epigenetic characteristic of the longevity in this population.

### B Lymphocytes

Considering the role of epigenetic mechanisms in B cell differentiation and function, age-associated epigenetic changes could be the responsible for the decline of humoral immunity in elderly individuals. Loss of function of B cells and their progenitors, reduction in the immunoglobulin diversity and affinity, and shifts in the proportion of naïve and antigen-experienced peripheral B cells subpopulation are characteristics of immune aging (216, 217). Hematopoietic stem cells (HSCs) lose their capacity to differentiate with age, and epigenetic alterations are important contributors to this change. Aged mice present HSCs with an aberrant gene expression profile because of epigenetic deregulation (218). Defects on both B-lineage commitment and transit through early development stage are observed during aging (219).

## Conclusion and Perspectives

Considerable progress has been made in understanding epigenetic alterations involved in aging over the recent years. Most of recent knowledge about epigenetics and aging is almost lack regarding immune aging. Many gaps and questions are still open and should be deeply investigated in this area. However, there are also important challenges and limitations in this study. For example, several analyses in this field use samples contained mixed cell types, instead isolated cells, which may bring confusion in the data interpretation. The dynamic nature of the immune system *per se* becomes challenging to study plastic molecular alterations involved in immune responses. It remains to be determined which epigenetic changes are causally related to aging process, and how they cause immune aging. Future studies are needed to determine the overlapping epigenetic signatures between immune aging and age-related immune diseases.

**Figure 1:**
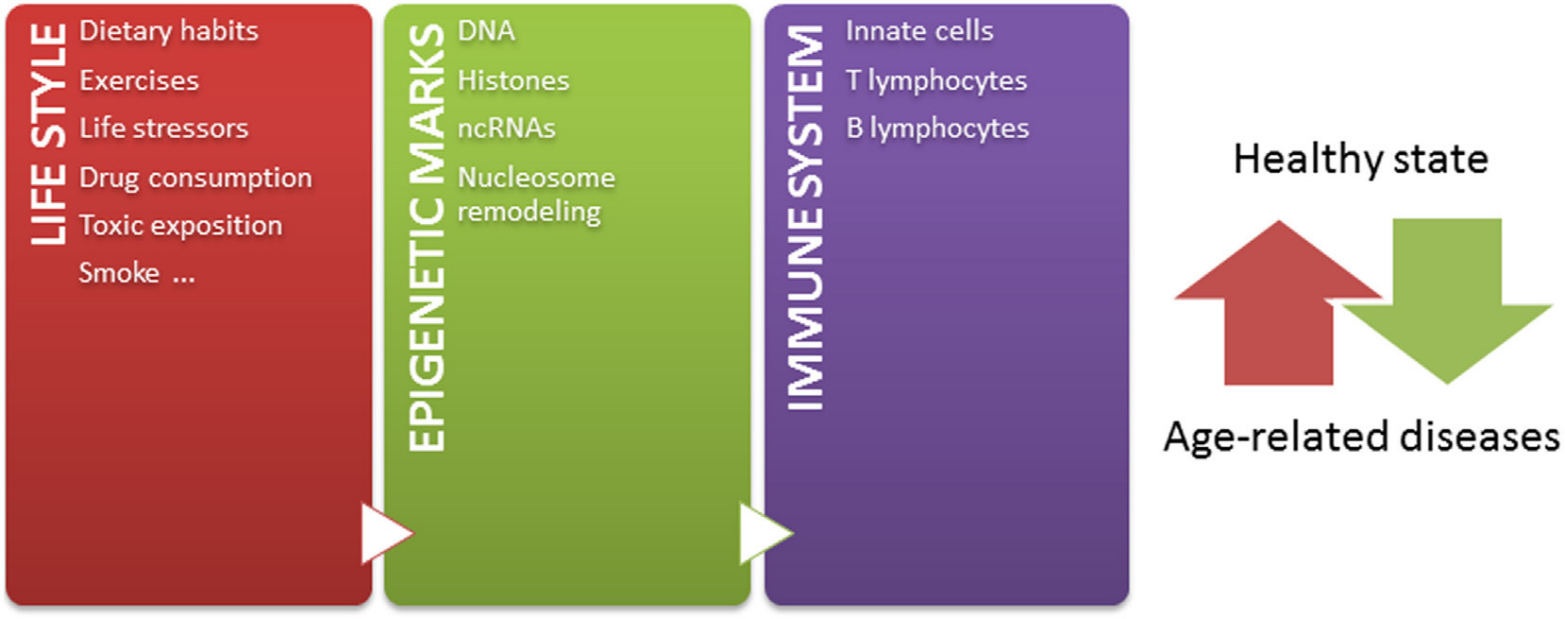
The epigenetic landscape, modulated by intrinsic and extrinsic factors, regulates immune system and contributes to define a healthy or pathological state. Our life style, including nutritional habit, exercises, life stressor, drugs, toxics exposition, and smoke, dynamically sculpts our epigenome along lifetime. The immune system is one of the systems in which functions are importantly regulated by epigenetic mechanisms. In this way, the accumulation of abnormal epigenetic marks in immune cells might contribute to the development of age-related diseases.

Independently of these challenges, and taking into account that: (1) epigenetic mechanisms modulate chromatin states, defining gene expression profiles, (2) epigenetic mechanisms play crucial roles in the development and function of immune system, (3) a tightly regulated functioning of immune system is necessary to maintain a healthy state, (4) the environment modifies epigenetic marks throughout lifetime, and (5) epigenetic marks are potentially reversible, the knowledge about how the environment modulates the immune system by epigenetic mechanisms contributing to age-related diseases may lead to the design of novel strategies for prevention and therapeutics. Since some age-related epigenetic alterations are similar across a range of tissues (52, 220), these alterations could be also potentially used as biomarkers for aging-related disease phenotypes in biological samples, such as blood or saliva. But most importantly, considering that both intrinsic and external factors modify epigenetic marks throughout life, it is important to have in mind that healthier lifestyle may be still the most effective way to prevent diseases later in life (Figure 1) (221). In conclusion, huge efforts should be undertaken to better understand the relation among epigenetics, immune aging and age-related diseases, in order to define interventions in the lifestyle able to modulate our epigenome for a healthy aging.

## Author Contributions

The author confirms being the sole contributor of this work and approved it for publication.

## Conflict of Interest Statement

The author declares that the research was conducted in the absence of any commercial or financial relationships that could be construed as a potential conflict of interest.

## Abbreviations

ncRNA, noncoding RNA; 5mC, 5-methylcytosine; DNMT, DNA methyltransferase; SAM, *S*-adenosylmethionine; TET, ten-eleven translocation; 5hmC, 5-hydroxymethylcytosine; 5fC, 5-methylformylcytosine; 5caC, 5-caboxylcytosine; PTM, posttranslational modification; 8-OHdG, 8-hydroxy-2′-deoxy-guanosine; DC, dendritic cell; NK, natural killer; APC, antigen-presenting cell; IFN-γ, interferon-gamma; IL, interleukin; MIP-1α, macrophage inflammatory protein 1-alpha; LPS, lipopolysaccharide; PRR, pattern recognition receptor; DAMP, damage-associated molecular pattern; PAMP, pathogen-associated molecular pattern; TCR, T cell receptor; NFAT, nuclear factor of activated T cells; MHC, major histocompatibility complex; CIITA, class II transactivator; Treg cell, regulatory T cell; TGFβ, transforming growth factor β; RORC, RAR-related orphan receptor C; FOXP3, forkhead box P3; Pax5, paired box 5; AID, activation-induced cytidine deaminase; Ezh2, enhancer of zeste homolog 2; MOZ, monocytic leukemia zinc finger protein; HPC, hematopoietic progenitor cell; TNF-α, tumor necrosis factor-alpha; TLR-2, toll-like receptor 2; CRAT, carnitine *O*-acetyltransferase; F3, coagulation factor III; CRP, C-reactive protein; EWAS, epigenome wide association studies; HSC, hematopoietic stem cell.
